# The More the Merrier—Complexity in Long Non-Coding RNA Loci

**DOI:** 10.3389/fendo.2017.00090

**Published:** 2017-04-25

**Authors:** Christian Ziegler, Markus Kretz

**Affiliations:** ^1^Institute of Biochemistry, Genetics and Microbiology, University of Regensburg, Regensburg, Germany

**Keywords:** long non-coding RNAs, non-coding RNA, alternative splicing, alternative polyadenylation, bifunctional RNA

## Introduction

### Long Non-Coding RNAs (lncRNAs)—From Ignorance to Importance

One of the long-standing principles of molecular biology has been that DNA functions as a template for transcription of messenger RNAs, which are eventually translated into a protein. Thus, proteins were seen as the main mediators of nearly all aspects of cell and tissue function. However, this perception started changing rapidly when high-throughput sequencing platforms became available, unraveling that more than two-thirds of the human genome are transcribed into RNA but only <2% of transcripts encode proteins (1, 2). Thus, the majority of the transcriptome falls into the category of non-coding RNAs (ncRNAs). These include long-known and well-characterized classes of ncRNAs with basic cellular housekeeping functions such as translation (transfer RNAs and ribosomal RNAs), splicing (small nuclear RNAs), or RNA editing (small nucleolar RNAs) (3, 4). Furthermore, short regulatory ncRNAs (20–30 nt in length) including microRNAs, piwi-associated RNAs, or endogenous short-interfering RNAs are highly conserved among species and have been proven to be crucial regulators of gene expression (5–7). Apart from these rather well-studied ncRNAs, the more recently identified class of lncRNAs has gained increasing scientific interest over the past years, and we are only beginning to appreciate their significance in a multitude of cellular processes and their complex modes of action (8, 9).

The original classification of lncRNAs is based on a length of at least 200 nt and lack of protein-coding potential. lncRNAs can be spliced, capped, and/or polyadenylated and localize either to the nucleus or the cytoplasm of the cell (1, 10). Interestingly, several lncRNAs were recently shown to act as templates for small peptides, and a number of mRNAs appear to adopt additional non-coding functionality (11–16). These observations suggest that classification of RNAs based on protein-coding potential might not in all cases be sufficiently exhaustive.

In contrast to mRNAs, lncRNAs generally show less primary sequence conservation among species, contain fewer but longer exons, and exhibit an intriguingly cell type-specific expression (8, 9). In addition, lncRNAs have been proven essential for processes such as cellular differentiation and progenitor cell regulation, epigenetic imprinting, X-chromosome inactivation, promoter-specific gene regulation, and nuclear import (17–25). Moreover, aberrant lncRNA expression has been linked to several diseases, including many types of cancer, highlighting their functional relevance during these diverse processes and rendering lncRNAs a captivating and novel research field (26–30). The frequently observed high level of complexity and diversity of gene loci, however, can significantly complicate functional characterization of lncRNAs. Hence, careful analysis of lncRNA function should start with a close characterization of its genomic locus, especially if the lncRNA is not yet characterized or its gene locus not well annotated, to lower the chance of drawing wrong conclusions and dissipating time and money. Below, we will illustrate mechanisms of lncRNA isoform generation using selected examples, introduce several approaches for lncRNA locus studies, and discuss potential pitfalls in investigating lncRNA loci.

## Diversity in lncRNA Loci and Experimental Strategies to Explore Them

### The Genomic Landscape of lncRNA Loci

#### Origins for lncRNAs within the Genome

**Figure 1:**
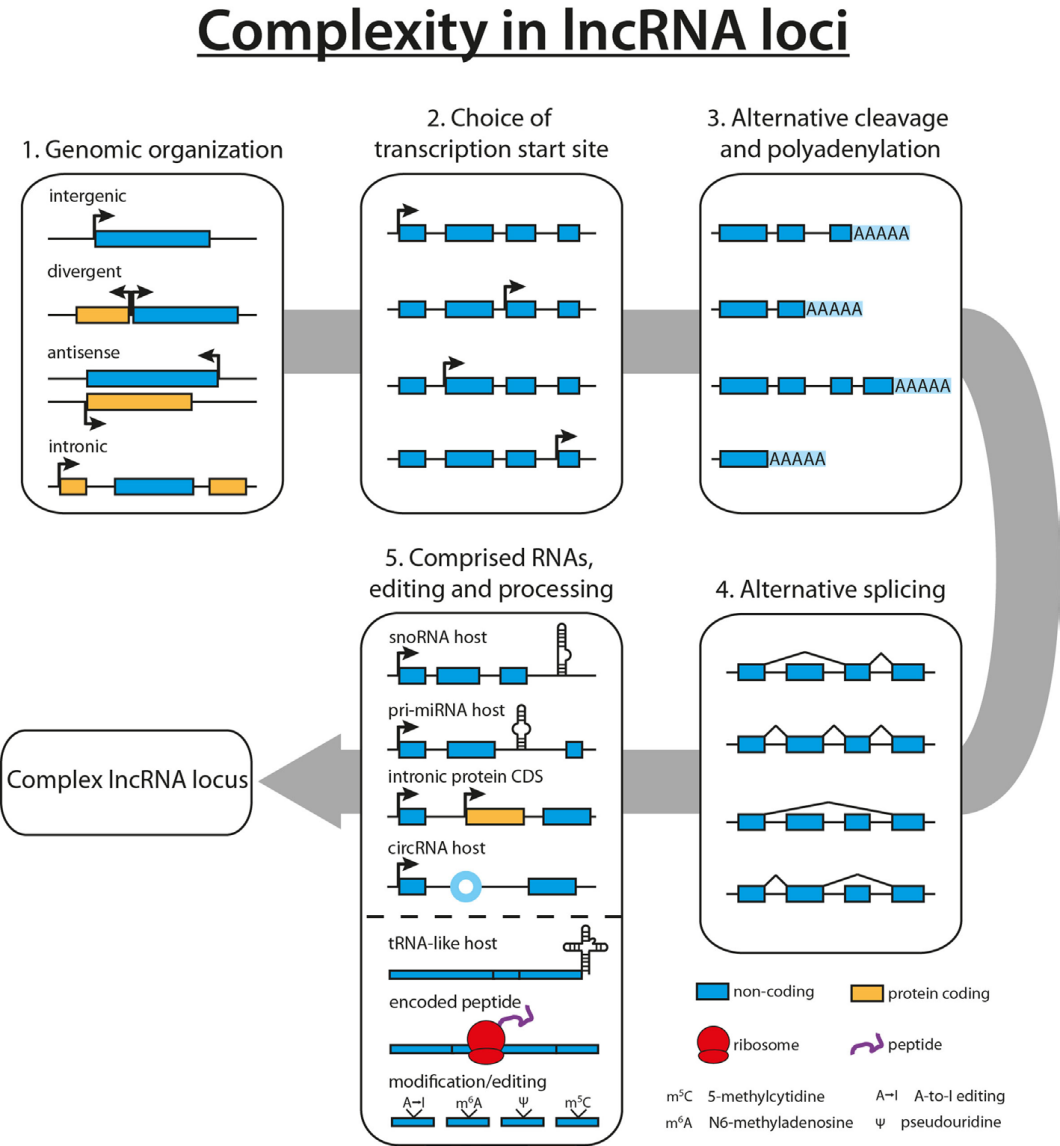
**Complexity in long non-coding RNA (lncRNA) loci**. Diversity in lncRNA loci originates from the genomic organization of the lncRNA (1). A plethora of lncRNA isoforms arises from the combination of multiple transcription start sites (2), alternative cleavage and polyadenylation sites (3), as well as alternative splicing events (4). Finally, lncRNAs have been shown to harbor other non-coding RNAs such as snoRNAs, miRNAs, or tRNAs or to contain intronic protein-coding genes, increasing the potential complexity of lncRNA loci (5). In addition to diverse loci, lncRNAs can give rise to tRNA-like molecules, encode small peptides, or are subject to RNA modification and editing events (5).

With the lncRNA field still being in its infancy, novel lncRNAs are detected in human cells and tissues on a regular basis, resulting in several thousand predicted human lncRNAs to date (8, 31, 32). Studies focusing on the complexity within lncRNA loci revealed up to 40 different isoforms for the lncRNA PCBP1-AS1, with an average of 2.3–3.9 different isoforms per locus, accentuating the necessity to complement the functional analysis of an lncRNA with a thorough characterization of its gene locus (8, 9, 33). Scattered all over the genome, lncRNA genes can be found far away from other annotated genes, or lncRNAs can emerge in the opposite direction of a neighboring gene locus (divergent). In addition, several lncRNAs were found to reside within an intron (intronic) or being the antisense transcript of a protein-coding gene, thus sharing the same gene locus (see Figure 1) (34).

#### Expanding the Picture—Translated lncRNAs and Hosts for ncRNAs

In addition to being part of another transcriptional unit, lncRNAs themselves can harbor protein-coding genes or other ncRNAs such as circular RNAs, tRNAs, miRNAs, and snoRNAs (8, 35–39). A prime example for this complexity is the lncRNA GAS5, which hosts 10 C/D box snoRNAs, five of which can be further processed to piRNAs (40, 41).

Despite their classification as long “non-coding” RNAs, several studies showed that lncRNAs can be translated into small peptides and are associated with ribosomes, further increasing the complexity within lncRNA loci (14, 42, 43). In addition, mRNAs can harbor regulatory RNA functions such as miRNA sponges, transcription elongation, or translational control (12, 13, 16). One of the earliest observations of these bifunctional RNAs is that both SRA1 and its protein product SRAP can act as transcriptional coactivators of nuclear receptors (11, 44). More recently, the peptide DWORF was identified in the lncRNA LOC100507537, and many additional putative peptides are predicted to arise from lncRNAs (15, 42, 43). On the other hand, Bánfai et al. correlated tandem mass spectrometry data with RNA sequencing (RNA-seq) data (both generated in two different cell lines by ENCODE) and found over 90% of GENCODE lncRNAs to be unlikely to encode a peptide. This is in accordance to a similar approach by Gascoigne et al., suggesting that the majority of lncRNAs likely is truly non-coding (45, 46). Thus, experimental validation of a bioinformatically predicted small peptide is needed to verify translation, stability, and functional relevance.

### Front to Back—Diversity Originating in lncRNA Ends

Diversity within a lncRNA locus is not solely reflected by overlapping or embedded transcripts within a lncRNA but also the transcription initiation and termination sites can vary. Correspondingly, almost two different 3′ ends can be found for each transcriptional start of a given lncRNA (47, 48). One cause for alternative 3′ ends is alternative cleavage and polyadenylation (APA). Roughly, 70% of human and mouse genes undergo APA, and many lncRNAs exhibit alternative polyadenylation sites upstream of the most 3′ exon, whereas for mRNAs, alternative polyadenylation sites are often located within the last exon (49, 50). Interestingly, 15–45% of the conserved elements in lncRNAs are located behind the first polyadenylation site, suggesting a switch in lncRNA function regulated by APA (49). Thus, it is not surprising that lncRNAs can be guided to different cellular compartments by alternative cleavage and polyadenylation as reported for CCAT-1 (51).

Besides alternative polyadenylation, 3′ processing of lncRNAs has been described, further expanding the potential diversity of lncRNA isoforms generated within the cell. As an example, the lncRNAs MALAT1 and NEAT1 feature a tRNA-like structure at their 3′ end, which is subject to RNAse P cleavage, resulting in a stable RNA triple-helix at the 3′ end of both lncRNAs, which serve as compensatory poly-A tails (52–54).

Similar to mRNAs, lncRNAs exploit the usage of alternative transcription start sites (55, 56). For the lncRNA Tsix, which is involved in the process of X-chromosome inactivation, two different transcription start sites have been identified. Correspondingly, the gene locus of the lncRNA SOX2OT has at least two promoter regions (57, 58).

By using both alternative polyadenylation and transcription start sites, a multiplicity of lncRNAs originating from the DM1-AS locus has been reported, which is even further increased through alternative splicing events (59).

### Alternative Splicing of lncRNAs

Despite functioning in the regulation of RNA splicing, lncRNAs too can be alternatively spliced, which presumably alters their function within the cell (60, 61). One notable example is the lncRNA GNG12-AS1. Splicing of GNG12-AS1 results in a total of 38 different isoforms with up to 10 exons. Furthermore, cohesin has been identified as a splice regulator of GNG12-AS1, evoking the idea of tight splicing regulation to be crucial for maintaining the isoform-specific functions of GNG12-AS1 (39). Another example for the complexity of the human transcriptome through alternative splicing can be found for the lncRNA HOTAIR, which can act as a molecular scaffold: the 5′ end of HOTAIR binds the polycomb repressive complex 2 (PRC2), and the 3′ end interacts with the histone demethylase LSD1 (62). By bringing these two chromatin modifying complexes in close proximity and guiding them to target chromatin, HOTAIR mediates epigenetic silencing of the HOXD locus, thus leading to increased cancer invasiveness and metastasis (19, 20). Through alternative splicing, the PRC2-binding domain of HOTAIR can be removed, potentially changing the functionality of this lncRNA.

Along these lines, another study focused on the transcriptome of hepatocellular carcinoma (HCC) patients. As a result, Zhang et al. found that in addition to differential expression, lncRNAs also displayed alternative splicing in HCC specimens compared to controls, suggesting a potential role for those splice variants as biomarkers and therapeutic targets for HCC (63). Taken together, alternative lncRNA splicing may alter the function of a given lncRNA.

### Strategies and Drawbacks in Studying lncRNA Loci

Given the examples above, which illustrate multiple means of RNA isoform generation, we can assume that our current knowledge of diversity within gene loci in general is far from being complete, and current annotations are not always exhaustive. Accordingly, comprehensive analysis of gene loci might in many cases be necessary to enable accurate functional and mechanistic investigation of the resulting isoforms. Below, we will discuss approaches to identify expressed lncRNA isoforms and further explore a given lncRNA locus.

The gold standard in elucidating lncRNA expression and isoform discovery on a large scale is RNA-seq. In general, this sensitive method requires significant bioinformatical expertise, especially when investigating lncRNA isoforms or alternative splicing [for a review, see Ref. (64, 65)]. Nevertheless, there are some useful tools that require only limited bioinformatical knowledge and are publicly available, which can be seen as a starting point for isoform prediction. Employing genome browsers such as UCSC allows direct uploading of aligned RNA-seq data from the cell or tissue type of interest. This enables locus-specific mapping and comparison with potentially annotated lncRNA isoforms or publically available histone mark occupancy data as well as further expression tracks to support predictions of lncRNA isoforms from genomic areas devoid of any annotation (66). When inspecting RNA-seq reads, one should keep in mind that many lncRNAs are rather low expressed and exhibit a tissue-specific expression pattern, so adequate sequencing depth is required for isoform analysis (8, 9). Attention should also be attributed to the employed library preparation technique. Using oligo-dT-based enrichment strategies will result in loss of the non-polyadenylated lncRNA population within the transcriptome. Usage of non-poly-A selective library preparation methods can circumvent this problem, but at the cost of sequencing depth and the requirement for additional means of rRNA removal (67). Furthermore, strand-specific library preparation protocols offer the possibility to distinguish between sense and antisense transcripts (68).

Even though RNA-seq is very sensitive and bioinformatical tools are constantly improving, the experimental validation of potential isoforms employing methods such as northern blot or rapid amplification of cDNA ends (RACE) is still required to complement the bioinformatic predictions. With RACE, the RNA ends can be deciphered; however, most approaches utilize a 3′ poly-A tail, circumventing the detection of non-polyadenylated transcripts (69). For 5′ RACE, protocols exploiting the 5′ cap have been established, ensuring only detection of intact transcripts rather than also picking up potential degradation products (70). Identification of uncapped transcripts on the other hand requires classic 5′ RACE approaches, which might result in an overestimation of isoforms and transcription start sites (70). Supplementing RACE, cap analysis gene expression (CAGE) may unravel the 5′ end of capped RNAs, and recent modifications to the original protocol such as nanoCAGE or nAnT-iCAGE have been developed to work with minimal starting material and exclude bias from PCR amplification or tag cleavage (71, 72). Before performing own CAGE analysis, the recently published FANTOM5 data set can be mined for the occurrence of 5′ start sites for a given locus (73).

Once a pool of potential isoforms has been established, northern blots can be used to verify their presence and predicted length (74). Moreover, the abundance of approved isoforms can be determined by (q)RT-PCR using isoform-specific primer sets (75). Overall, none of the mentioned techniques alone might be sufficient for verification and mapping of multiple isoforms because each technique not only has certain strengths but also has weaknesses. However, using these methods as complementary approaches and compiling insights from all analyses may allow isoform prediction as well as isoform verification.

## Concluding Remarks

With new lncRNAs being continuously identified, this exciting research field is rapidly growing. In the hunt for new functions and mechanisms, close attention has to be paid to the wealth of lncRNA isoforms and their potential to being processed to other ncRNAs or translated into small peptides to discover new facets of lncRNAs. Recently, the presence of lncRNA modifications and lncRNA editing has been reported and associated with structural and functional changes, increasing the variety of lncRNAs (52, 53, 76–78). For example, m^6^A on position 2,577 of MALAT1 was found to alter its secondary structure, resulting in tighter binding of heterogeneous nuclear ribonucleoprotein C (77, 78).

Within this article, we highlighted the complexity of lncRNA isoform generation and outlined approaches for lncRNA isoform detection and their drawbacks, which should be rather seen as impulses than an exhausting discussion and motivate researchers to move forward into this very intriguing and challenging field of study.

## Author Contributions

CZ and MK drafted and revised the manuscript.

## Conflict of Interest Statement

The authors declare that the research was conducted in the absence of any commercial or financial relationships that could be construed as a potential conflict of interest.
